# Drinking and Driving among Recent Latino Immigrants: The Impact of Neighborhoods and Social Support

**DOI:** 10.3390/ijerph13111055

**Published:** 2016-10-28

**Authors:** Mariana Sanchez, Eduardo Romano, Christyl Dawson, Hui Huang, Alicia Sneij, Elena Cyrus, Patria Rojas, Miguel Ángel Cano, Judith Brook, Mario De La Rosa

**Affiliations:** 1 Center for Research on U.S. Latino HIV/AIDS and Drug Abuse (CRUSADA), Robert Stempel College of Public Health and Social Work, Florida International University, 11200 SW 8th Street, AHC5-421, Miami, FL 33199, USA; cdaws011@fiu.edu (C.D.); huanhu@fiu.edu (H.H.); asnei001@fiu.edu (A.S.); ecyru002@fiu.edu (E.C.); proja003@fiu.edu (P.R.); mcanojr@fiu.edu (M.Á.C.); delarosa@fiu.edu (M.D.L.R.); 2 Pacific Institute for Research and Evaluation, 11720 Beltsville Drive Suite 900, Calverton, MD 20705-3111, USA; romano@pire.org; 3 Department of Epidemiology, Robert Stempel College of Public Health and Social Work, Florida International University, 11200 SW 8th Street, Miami, FL 33199, USA; 4 New York University School of Medicine, NYU Langone Medical Center 215 Lexington Ave, New York, NY 10016, USA; judith.brook@nyumc.org

**Keywords:** drinking and driving, alcohol, Latino/a immigrants, acculturation stress, neighborhoods

## Abstract

Latinos are disproportionately impacted by drinking and driving arrests and alcohol-related fatal crashes. Why, and how, these disparities occur remains unclear. The neighborhood environments that recent Latino immigrants encounter in their host communities can potentially influence health behaviors over time, including the propensity to engage in drinking and driving. This cross-sectional study utilizes a sample of 467 documented and undocumented adult recent Latino immigrants in the United States to answer the following research questions: (a) How do neighborhood-level factors, combined with social support, impact drinking and driving risk behaviors?; and (b) Does acculturative stress moderate the effects of those associations? Results indicate neighborhood-level factors (informal social control and social capital) have protective effects against drinking and driving risk behaviors via the mediating mechanism of social support. Acculturative stress moderated associations between neighborhood informal social control and social support, whereby the protective effects of informal social control on social support were not present for those immigrants with higher levels of acculturative stress. Our findings contribute to the limited knowledge of drinking and driving among Latino immigrants early in the immigration process and suggest that, in the process of developing prevention programs tailored to Latino immigrants, greater attention must be paid to neighborhood-level factors.

## 1. Introduction

In the United States, alcohol-related motor vehicle crashes represent a major public health problem. Latinos in the U.S. are particularly impacted by drinking and driving health disparities [1]. As defined by the National Institutes of Health, health disparities are differences in the prevalence, incidence, mortality, and burden of diseases and other deleterious health conditions existing among specific population groups [2]. Compared to non-Latino Whites, Latinos have consistently had a higher prevalence of being involved in alcohol-related crashes—42% of fatally injured Latino drivers, compared to 29% of White drivers, have blood alcohol content (BAC) levels over 0.08 g/dL [3].

Most existing research on drinking and driving among Latinos has focused on individual-level variables. The studies suggest that these alcohol-related disparities may be exacerbated by socio-cultural factors, such as level of acculturation—potentially influencing adherence to traffic laws [4,5]. Certain Latino subgroups may be particularly susceptible to drinking and driving. For instance, recent Latino immigrants could be at higher risk for drinking and driving due to a failure to fully understand the risks associated with drinking and driving as a result of cultural norms from their countries of origin, including lax enforcement of drinking and driving laws [6,7].

A recent study conducted by Romano et al. was among the first to examine drinking and driving in a sample of documented and undocumented recent Latino immigrants in the United States [8]. Findings revealed that, compared with documented Latino immigrants, undocumented Latino immigrants are more likely to binge drink, less likely to understand Driving Under the Influence (DUI) laws, and less likely to perceive the risks associated with drinking and driving—three factors largely associated with high DUI rates. Despite facing these risk factors, relatively low drinking and driving rates were found among undocumented immigrants; low drinking and driving rates among undocumented immigrants were due to their limited driving rates and their desire to avoid law enforcement officers and remain “under the radar”. Regardless of the causes, the noted variability in drinking and driving rates among recent Latino immigrants suggests an opportunity to engage this population in culturally-appropriate interventions aimed at reducing existing disparities in alcohol-related car crashes [8].

The development of culturally-appropriate interventions requires moving research from individual-level factors toward a broader examination of how neighborhoods may impact drinking and driving behaviors in recent Latino immigrants [9]. Over the past twenty years, there has been an emerging interest in the impact of neighborhoods on health outcomes. This trend parallels the increasing attention, not only to individual characteristics and behavior, but also to the context in which individuals live when examining variances in health risk behaviors across populations [10]. Group social processes at the neighborhood-level (e.g., social capital, informal social control) and the physical aspects of neighborhoods are associated with health outcomes, including those associated with alcohol use [10,11]. A longitudinal study of recent Latino immigrants conducted by Sanchez et al. found decreases in pre- to post-immigration alcohol use in this population [12]. The investigators posited that factors related to social control (e.g., prohibition of drinking and driving) that may not exist in the country/region of origin may serve as a way to monitor and direct alcohol-related behaviors toward what is considered acceptable by the host society.

Given that place of residence is often linked to socio-economic status and ethnicity, neighborhood characteristics have the potential to significantly contribute to risk and protective factors influencing health disparities among minorities. As such, neighborhood-level interventions have the potential to significantly reduce health disparities, including the disproportionate impact of alcohol-related crashes in Latino communities in the United States. The present study seeks to understand the mechanisms whereby neighborhood factors impact drinking and driving risk behaviors among recent Latino immigrants. This knowledge can provide the foundation for the development of culturally-relevant community-based interventions targeting this population.

### 1.1. Theoretical Framework

The conceptual framework for the present study draws upon concepts from four theories. First, we rely on social capital theory, which focuses on the value of social networks as well as the norms of reciprocity and trustworthiness that arise from them. Several definitions for social capital exist—most definitions focus on the importance of social connections between individuals within neighborhoods and communities [13,14]. Social capital may be obtained through participation in religious institutions, recreational participation, workplace connections, and informal social ties. Although social capital is usually measured at the individual level, Putnam acknowledges that formal neighborhood institutions have the capacity to foster individual-level networking that leads to social capital [15]. For instance, the presence of religious institutions may increase religious participation among a neighborhood’s residents, thereby increasing tangible and intangible social connections among its members. Actively participating in schools, parks, and neighborhood associations can also increase social connections among neighborhood residents. These types of institutions symbolically and physically represent neighborhood characteristics that promote social networking, which can, in turn, increase access to social support [15,16].

Neighborhoods with high levels of social capital and social support may also have greater neighborhood informal social control. According to the social disorganization theory, informal social control is realized via neighbors’ willingness to intervene on behalf of the common good [17]. Instead of relying on formal controls, such as police and the legal system, residents in socially-cohesive neighborhoods are likely to execute informal social control to achieve public order; for example, residents that voluntarily monitor playgroups among children. The social disorganization theory considers informal social control and social cohesion as the mechanisms behind the association between neighborhood conditions and behavioral problems. Using data from the Survey of New York City of middle/high school students and the 1990 Census, Seidman et al. reported that negative neighborhood experience (e.g., low social cohesion) is associated with increased risk of antisocial behaviors, such as alcohol use [18]. In a recent study, Worrall et al. reported that informal social control also moderates the effects of certainty and severity of punishment on offending likelihood [19]. That is, certainty and severity of punishment are more likely to deter offenders only when high informal social control is present.

Our study is also informed by segmented assimilation theory. This framework asserts that the process of immigrant assimilation to a new country may lead to various forms of cultural adaptation [20]. Acculturation trajectories leading to upward assimilation are associated with higher levels of social capital and an immigrant-receiving community that potentially insulates some immigrants from the negative influence of deleterious risk behaviors of the dominant society [21]. Downward assimilation is associated with economic hardships, a lack of resources, and exposure to discrimination, circumstances that can lead to acculturative stress. Segmented assimilation theory also emphasizes “the social context in which [immigrants] are received in America” (p. 13) [22]. Previous research has demonstrated the negative impact that residing in neighborhoods with low social cohesion can have among immigrants [21]. Conversely, residing in Latino ethnic enclaves may provide immigrants with a greater sense of ethnic identity and connectedness, which could serve to guard community members from acculturative stressors and its associated negative outcomes (e.g., alcohol use). Previous research has found concentrations of recent immigrants in a neighborhood to be a protective influence against substance abuse [23]. Overall, segmented assimilation theory highlights the tendency for recent immigrants to adapt to the dominant host culture—*unless* ethnic and social resources operate to provide alternate patterns of cultural adaptation [20]. The present study proposes the intersectionality of segmented assimilation, social capital, and social disorganization theory to assume increased trajectories of drinking and driving over time among recent Latino immigrants unless bolstered and supported by regular access to protective neighborhood-level factors (i.e., social capital and neighborhood informal social control).

Lastly, we utilize acculturative stress theory to inform our research model. Recent Latino immigrants often face numerous stressors when arriving to the United States. Acculturation-related stressors include language barriers, discrimination, loss of social support and difficulties assimilating to the beliefs and values of the dominant culture [24]. This form of stress has been linked with numerous deleterious mental and physical health outcomes including increased alcohol-use problems among Latinos [25]. The present study posits that the protective direct and indirect effects of social capital, neighborhood informal social control and social support on drinking and driving risk behaviors will be weaker among recent immigrants experiencing high levels of acculturative stress.

### 1.2. Research Aims

The overarching aims of this study are to elucidate the mechanisms whereby neighborhood-level factors impact DUI risk behaviors among recent adult Latino immigrants by: (a) testing if social support mediates the effects of neighborhood informal social control and social capital on DUI risk behaviors; and (b) determining if acculturative stress moderates the effects of neighborhood informal social control and social capital on DUI risk behaviors.

The following hypotheses were developed: H1: Higher levels of informal social control and social capital will be directly associated with higher levels of social support. H2: Higher levels of social support will be directly associated with lower rates of drinking and driving risk behaviors. H3: Neighborhood informal social control and social capital will have indirect effects on drinking and driving risk behaviors via the mediating influence of social support. H4: Acculturative stress will moderate the direct effects of informal social control and social capital on social support, as well as the direct effects of social support on drinking and driving risk behaviors. We anticipate positive associations between informal social control/social capital and social support will be weaker among Latino immigrants experiencing higher levels of acculturative stress. We hypothesize protective effects of social support on drinking and driving risk behaviors will also be weaker among Latino immigrants experiencing higher levels of acculturative stress.

### 1.3. Neighborhoods and Alcohol Use

Neighborhood-level factors, such as social capital and informal social control, can significantly impact the prevalence of substance abuse [26]. In the United States, immigrant-receiving communities supply immigrants with social capital that can potentially influence health behavior over time, including alcohol use-related trajectories [27]. Social capital involves norms of reciprocity and trust within a community, including community participation, engagement, organization, and social cohesion [14]. Additionally, residing in neighborhoods with high neighborhood social cohesion can foster “loose” interpersonal connections that potentially lead to positive structural benefits, including better access to social and health services that can mitigate health-compromising behaviors such as substance use among Latinos [28,29]. Conversely, neighborhoods with poor physical infrastructure, low social cohesion, and inadequate health care resources have been found to place residents at risk for alcohol misuse as well as other health risk behaviors [30,31]. The present study examines how various levels of neighborhood social capital and informal social control influence drinking and driving risk behaviors among recent adult Latino immigrants.

### 1.4. Social Support

Previous studies have shown that social support mediates the effects that the neighborhood environment has on the well-being of Latino immigrants [32]. The environmental conditions in which people live may foster social connections which, in turn, affect individuals’ tangible and intangible resources, making them more likely to engage in health-promoting behaviors. The immigration process often disrupts social networks. Less time in the U.S. has been associated with decreased social ties among Latino immigrants [33]. However, social support has been found to be an important mitigating factor in buffering acculturative stress among Latino immigrants [34]. Investment in social relations facilitates the flow of information [35], making the acculturation process less difficult and stressful. In a study by Concha et al., acculturative stress was negatively associated with various forms of social support among recent Latino immigrants [26]. Cano et al. also found that social support consistently reduced the deleterious effect of acculturative stress on alcohol use severity in the same sample [36]. These findings reaffirm that Latino immigrants rely on others for tangible and emotional social support during stressful events related to the acculturation process [37,38,39].

### 1.5. Acculturative Stress

Acculturation is a multidimensional process by which changes in individual norms and behavior occur over time as a result of contact with a new culture [40]. Ample evidence suggests that alcohol use is greater among Latino immigrants with higher levels of acculturative stress [41,42,43]. In particular, acculturative stressors related to the context of the immigrant-receiving community—such as language barriers, perceived feelings of marginalization/discrimination, and undocumented legal status—can impact alcohol use patterns among recent Latino immigrants [36]. While the link between acculturation and drinking behaviors has been widely examined, there is a paucity of research on the cultural influences of DUI risk behaviors among documented and undocumented Latino immigrants. Although some evidence has shown Latino drivers’ involvement in alcohol-related crashes to increase with higher levels of acculturation, particularly among women [44], to our knowledge, no clear examination of the cultural aspects of this phenomenon has been previously conducted.

## 2. Materials and Methods

### 2.1. Procedure

The Recent Latino Immigrant Study was the first prospective community-based cohort study to examine the pre- to post-immigration alcohol use trajectories of young adult recent Latino immigrants during their first three years in the United States. Study findings included associations between various social determinants (i.e., decreases in social capital, religious coping, and family cohesion) as potential mechanisms of pre- and post-immigration alcohol use [45,46,47]. A subsequent cross-sectional follow-up study examined drinking and driving behaviors in the sample 6–7 years post-immigration. The present study is based on this cross-sectional investigation.

Inclusion criteria for the original study included being a Latino, 18–34 years old, who had recently immigrated (i.e., within one year prior to the baseline assessment) to the United States from a Latin American country with the intention of staying in the United States at least two years beyond the baseline assessment. Given the scope of the study, immigrants from non-Spanish speaking countries of Latin America (Brazil, Suriname, Guyana) were not included in the sampling frame. Respondent-driven sampling (RDS) was the primary strategy whereby most of the sample was recruited. The initial wave of recruits (seeds) was asked to refer three individuals in his or her social network who met the study eligibility criteria. Seeds were recruited via flyers posted in neighborhoods with substantial Latino populations, during health fairs, and through community health centers. The depth of each chain was no more than three legs from the seeds. This technique is an effective strategy in recruiting participants from difficult-to-reach populations [48]. The study is distinguished by its success in reaching, accessing, and retaining the participation of undocumented immigrants [49].

The study’s research protocol was reviewed and approved on 10 September 2013 by the Institutional Review Board at Florida International University in Miami, FL, USA (# IRB-13-0361). A certificate of confidentiality was obtained from the National Institutes of Health to ensure maximum protection for research participants.

### 2.2. Measures

**Sociodemographics.** A demographics form assessed, in part, participants’ education level (1 = *less than high school*; 2 = *high school*; 3 = *some training/college after high school*; 4 = *bachelor’s degree*; 5 = *graduate/professional studies*), and income. For the present study, an income variable was computed by dividing total household income in the past 12 months by the total number of people dependent on that income.

**Documentation status.** U.S. documentation status was measured by 14 possible categories (e.g., temporary or permanent resident, temporary work visa, undocumented or expired visa). These categories were recoded into a dichotomous variable: documented = 1 or undocumented = 0.

**Alcohol use.** Alcohol use was assessed through the validated Spanish questionnaire version of the Alcohol Use Identification Test (AUDIT) [50]. The AUDIT is a 10-item screening questionnaire that contains three questions on the amount and frequency of drinking, three questions on alcohol dependence, and four questions on problems caused by alcohol. The scale indicated high reliability estimates in the present study (α = 0.91).

**DUI risk behaviors.** A dichotomous variable assessed DUI risk behaviors in the past twelve months. Items used were from the 2008 National Survey of Drinking and Driving [51]. Specifically, participants were queried with the following items: *In the past 12 months have you* (a) *driven within 2 h after drinking an alcoholic beverage?*; (b) *driven when you thought you were over the legal limit for alcohol and driving?*; and, (c) *ridden in a car with a driver that you thought may have consumed too much alcohol to drive safely?* If a respondent answered “yes” to any of these items, they were coded as 1 = DUI risk behaviors in past 12 months; all other were coded 0 = no DUI risk behaviors in past 12 months. 

**Neighborhood informal social control.** The Informal Social Control subscale of the Neighborhood Collective Efficacy Scale was used to assess this construct [17]. The scale contains 5 items and is in a 5-point Likert-type scale format ranging from (1) *very unlikely* to (5) *very likely*, with higher scores indicating more neighborhood informal social control. Example items include: (a) *If there was a fight in front of your house and someone was being beaten or threatened, how likely is it that your neighbors would break it up?*; (b) *If a group of neighborhood children were skipping school and hanging out on a street corner, how likely is it that your neighbors would do something about it?* In the present study, the scale indicated high internal consistency (*α* = 0.93).

**Social capital.** The Groups/Association subscale and Agency subscale from the Assets Inventory was used to assess social capital [52]. Combined, the subscales consist of 20 dichotomous (yes/no) items related to community resources used since their immigration to the United States. Resources include churches, informal social clubs, youth organizations, ethnic groups, community centers, recreation areas, schools, and libraries. A follow-up appraisal for each item asked if the resource was (1) *not at all helpful*; (2) *somewhat helpful*; or (3) *very helpful*. The sum of the frequency and appraisal scores was used to measure overall social capital. The scale has been previously utilized to measure various forms of social capital in this population [26,45].

**Social support.** The Medical Outcome Social Support Survey was used to measure social support [53]. The survey measures four dimensions of social support, including emotional/informational, tangible, affectionate and positive social interaction. The instrument contains 19 items set on a five-point Likert-type scale ranging from 1 = *none of the time* to 5 = *all of the time*, with higher scores indicating more social support. This Medical Outcomes Social Support Scale has been widely used and has been associated with numerous physical and mental health outcomes. In the present study, the total score of the four subscales of the Medical Outcomes Social Support Scale were utilized and demonstrated good internal consistency, (*α* = 0.99).

**Acculturative Stress.** The validated Spanish version of the immigration stress subscale of the Hispanic Stress Inventory Scale–Immigrant Version was used to measure acculturative stress [54]. This scale is a measure of psychosocial stress-event experiences for Latino immigrants. It has been widely used with Latino populations [55,56]. The instrument is in a five-point Likert-type scale format and the subscale used contains 18 questions. The participant reports whether or not he/she experienced a particular stressor. If the stressor was experienced, then a follow-up question is asked regarding the appraisal of how stressful that particular event was to the respondent (1 = *not at all* to 5 = *extremely*). Example items include: (a) *I felt guilty about leaving my family and friends in my home country*; (b) *Because of my poor English, it has been difficult for me to deal with day-to-day situations*; and, (c) *Because I am Latino, I have had difficulty finding the type of work I want.* Given the very high correlation between frequency and appraisal of stress in the present sample (*r* = 0.91), the sum of the immigration stress frequency and immigration stress appraisal scores was used to measure overall acculturative stress. 

Given that participants were recent Latino immigrants, all assessments were conducted in Spanish. For those measures without existing validated Spanish versions, we developed in-house Spanish translations. Specifically, the measures went through a process of (a) translation/back translation; (b) modified direct translation; and (c) checks for semantic and conceptual equivalence to ensure accurate conversion from English to Spanish [57]. In an effort to account for any within-group variability, the review panel conducting the modified direct translation consisted of individuals from various Latino subgroups representative of the Miami-Dade County population. This included representatives from the region’s largest immigrant groups such as Cuba, Colombia, Honduras, and Nicaragua, as well as representatives from smaller subsets of immigrant groups, including Dominican Republic, Peru, Bolivia, and Uruguay.

### 2.3. Data Analytic Plan

Initial data analyses included: (a) descriptive statistics for all predictor and outcome variables and (b) examining distribution properties of continuous variables for normality following Kline’s suggested cutoffs of absolute values of 3.0 and 8.0 for skewness and kurtosis, respectively [58]. A bivariate correlation matrix was computed to assess potential multicollinearity among key observed study variables. Tabachnick and Fidell suggest correlation coefficients of less than 0.70 between predictors to avoid multicollinearity [59]. Hypothesized covariates were gender, documentation status, age, education level, household income, and alcohol use. Spearman’s rho and Pearson correlations were used to analyze binary covariates (gender and documentation status) and continuous covariates (age, household income, and education), respectively. Chi-square analyses were used to test associations between binary covariates (gender and documentation status) and the binary outcome DUI risk behaviors.

### 2.4. Primary Analysis

**Figure 1:**
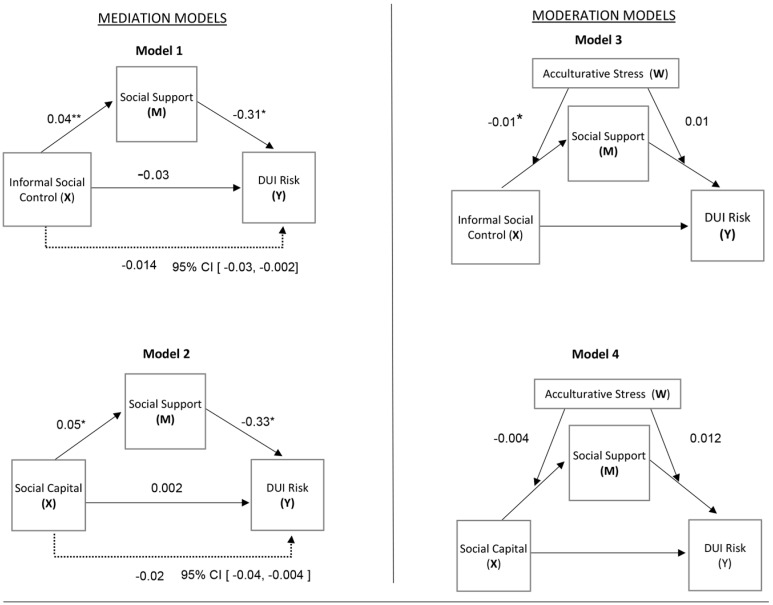
Results of tested mediation (**left panel**) and moderation (**right panel**) models; coefficients are provided in unstandardized values (b); * *p* < 0.05; ** *p* < 0.01; X: predictor; Y: outcome; M: mediator; W: moderator.

Four models with 10,000 bootstrap iterations were tested with the SPSS macro PROCESS v2.16 developed by Andrew F. Hayes, Columbus, Ohio, USA [60]. Figure 1 depicts the models tested in our analyses. Models 1 and 2 are mediation models. Model 1 examines the direct and indirect effects of neighborhood social control and social support on DUI risk behaviors. Model 2 tests the direct and indirect effects of social capital and social support on DUI risk behaviors. Models 3 and 4 are moderation models. Model 3 tests the interaction effects of acculturative stress on associations between neighborhood social control and social support on DUI risk behaviors. Model 4 examines the moderating effects of acculturative stress on associations between social capital and social support on DUI risk behaviors. 

For the moderation models, values for acculturative stress were calculated as the predicted values of the DUI risk behaviors for three distinct groups as follows: those who scored (a) at the mean; (b) one standard deviation above; and (c) one standard deviation below the mean on the predictor and moderator variables. It should be noted that PROCESS v2.16 only produces confidence intervals for unstandardized regression coefficients; therefore, results are presented in unstandardized values. All models were controlled for gender, documentation status, and alcohol use. Missing data were treated using list-wise deletion. Thus, 10 cases were deleted from the analyses.

## 3. Results

Table 1 presents descriptive statistics. Participants in the present study included recent adult Latino immigrants (*n* = 467). Participants were aged 23–40 when interviewed for this study. Approximately 45% of participants were men and 55% were women. The education levels reached were: post-graduate degree (1%), college degree (3%), some college (24%), high school or equivalent degree (50%), and less than high school (22%). Participants had relatively low income, with a reported average annual income of $19,962 (standard deviation = $16,524, median = $18,000). Approximately 84% of participants were documented immigrants, while 16% were undocumented.

The sample was fairly representative of the Miami-Dade County Latino immigrant community, which is 52.7% Cuban, 16.8% South American, 13.1% Central American, and 3.6% Other Caribbean. Present sample distributions by region of origin were: 43% Cuban, 28% South American, 28% Central American, and 1% Other Caribbean. Although the country/region of origin data for recent Latino immigrants in Miami-Dade County is not available, U.S. Census data indicated recent population increases in Miami-Dade County ranging from 102% to 117% among certain South American (e.g., Argentines and Venezuelans) and Central American (e.g., Hondurans and Guatemalans) Latino immigrant subgroups [61]. This population increase may explain the over-representation of South and Central Americans in the present study sample.

Table 1 also presents the means, standard deviations, and frequencies for all key study variables. Checks for normality indicated that neighborhood social capital was positively skewed. A square root transformation was conducted to obtain a normal distribution. All other variables were normally distributed.

Table 2 displays bivariate correlations for alel key variables in the present study. Analyses for potential covariates indicated significant correlations between documentation status and social support whereby participants with undocumented legal status reported less social support (*r* = 0.23, *p* < 0.001). Being male (*r* = −0.19, *p* < 0.001) and higher alcohol use (*r* = −0.23, *p* = 0.001) were associated with lower levels of social support. As anticipated, increased alcohol use was also associated with higher rates of DUI risk behavior (*r* = 0.22, *p* < 0.001). No significant differences were found by age, education, or income on the outcome variable. 

Bivariate correlation analyses also indicated that undocumented immigrants were more likely to reside in neighborhoods with lower level of informal social control (*r* = 0.20, *p* < 0.001), and have higher rates of alcohol use (*r* = −0.13, *p* = 0.004). However, no differences in levels of social capital were evident by documentation status. Compared to men, Latina immigrant women reported higher levels of informal social control (*r* = −0.10, *p* = 0.04), and informal social control, as well as lower rates of alcohol use (*r* = 0.25, *p* < 0.001).

### Primary Analyses

**Model 1:** We tested the mediating effect of social support on the associations between neighborhood informal social control and DUI risk behaviors (see Figure 1). Results indicate that predictor variables accounted for 14% of the variability in DUI risk behaviors. Higher levels of informal social control were directly associated with more social support (*b* = 0.04, *p* < 0.001). Greater social support was directly associated with lower rates of drinking and driving risk behaviors (*b* = −0.31, *p* = 0.03). Tests of conditional indirect effects suggest that informal social control had a statistically significant indirect effect on drinking and driving risk behaviors via social support (*b* = −0.014, 95% CI [−0.032, −0.002]). No direct effects of informal social control on the likelihood to engage in drinking and driving behaviors were found.

**Model 2:** We also tested the mediating effect of social support in the associations between social capital and DUI risk behaviors. Results indicated that predictor variables accounted for 12% of the variability in DUI risk behaviors. Higher levels of social capital were directly associated with more social support (*b* = 0.05, *p* = 0.003). Greater social support was directly associated with lower rates of drinking and driving risk behaviors (*b* = −0.33, *p* = 0.02). Tests of conditional indirect effects suggest that social capital had a statistically significant indirect effect on drinking and driving via social support (*b* = −0.02, 95% CI [−0.041, −0.004]). No direct effects of social capital were evident on the outcome variable.

**Model 3:** Figure 1 depicts the subsequent moderation model tested for interaction effects of acculturative stress in mediation Model 1. Results indicated a statistically significant interaction between informal social control and acculturative stress on social support (*b* = −0.01, *p* = 0.01), whereby the protective effect of informal social control on social support was not present for immigrants with high levels of acculturative stress.

**Model 4:** Lastly, we examined the moderating effects of acculturative stress in mediation Model 2. Results indicated no significant interaction effects.

## 4. Discussion

To our knowledge, the present study is the first to examine mechanisms whereby neighborhood-level factors impact drinking and driving risk behaviors among recent adult Latino immigrants. The key findings can be summarized as follows: neighborhood informal social control and social capital had protective effects against drinking and driving risk behaviors via the mediating mechanism of social support. Essentially, neighborhood factors provided the increased social support which, in turn, decreased risk behaviors among participants. Results from the moderation analyses indicated that acculturative stress only functioned as a modifier between informal social control and social support, whereby the protective effect of informal social control on social support was not present for those immigrants with high levels of acculturative stress. No other interaction effects were evident.

By testing the mediating role of social support, this study offers a possible explanation as to how neighborhood level factors function in curbing risk behaviors among recent Latino immigrants. In particular, the results add to the existing literature on the importance of social support in deterring drinking and driving in this population. These findings are in line with previous studies that have documented the manner in which neighborhood factors, combined with social support, can reduce health disparities in Latino communities. For instance, a community-based participatory prevention intervention titled *Poder es Salud*/Power is Health aimed at decreasing health disparities by increasing social capital within a Latino community [62]. Results showed increases in participants’ social capital was associated with higher levels of social support, as well as self-rated physical and mental health. In-depth interviews revealed that being a part of a group (e.g., a church) offered access to tangible and emotional social support and even provided opportunities to work collaboratively with group members to target specific public health issues plaguing their community [62].

Findings from the present study support the notion that future programs seeking to prevent drinking and driving among Latino immigrants should incorporate community-level strategies to increase aspects of social support, including civic participation and reciprocity among residents. Programs aimed at supporting recent Latino immigrants in their initial settlement phase should focus on expanding support resources by actively connecting new immigrants with local neighborhood supports.

The present study advances the understanding of how associations between acculturative stress and social support may impact health behaviors of Latino immigrants. Our findings reveal that acculturative stress weakened the positive effects of neighborhood factors on social support. Results from this study are consistent with previous research showing social support functions as a buffer to the negative effects that acculturative stress has on physical and mental health in Latino immigrant populations [63]. Overall, our findings indicate that acculturative stressors should be considered when developing culturally-relevant prevention programs with recent Latino immigrants.

However, the study results only partially support our hypotheses, as acculturative stress only had moderating effects between informal social control and social support. None of the other hypothesized moderating effects were found to be significant for acculturative stress. In part, the null findings may be related to the relatively low levels of acculturative stress reported in the sample. Given that participants had been residing in the United States for over six years at the time of assessment, stressors measured in the present study may not have been as relevant as during their first years in the United States. Previous studies have shown acculturative stress may be higher during the early years of immigration [64]. Another explanation may relate to the characteristics of our sample’s immigrant-receiving community. With Latino immigrants from a large variety of Caribbean and South and Central American countries, Miami-Dade County (MDC) is home to one the most diverse Latino populations of any U.S. city [65]. MDC has a highly multicultural environment that is equally supportive of U.S. and Latino cultural practices [66]. Latinos in this area tend to enjoy more political and economic advantages compared to other cities [67]. It is possible that well-established Latino immigrant-receiving communities with dense ethnic enclaves, such as those in MDC, may provide increased availability to culturally- and linguistically-congruent services, tangible and intangible support systems, and lower levels of discrimination that may impact associated health-compromising behaviors, such as drinking and driving.

Additionally, similar levels of social capital were reported for both documented and undocumented immigrants. This finding may also be related to the context of Miami-Dade County as an immigrant-receiving ethnic enclave. As anticipated, undocumented immigrants reported lower levels of social support, were less likely to reside in neighborhoods with lower rates of informal social control, and had higher rates of alcohol use. These results suggest that developing community-research partnerships with organizations that service undocumented Latino immigrants could enhance current outreach strategies and improve awareness of risks related to DUI in this population [68]. Previous studies examining drinking and driving among Latino immigrants have found street-level outreach interventions in recreation leagues and churches to be particularly effective in the Latino community [1].

Acculturative stress was associated with greater social capital. These findings are intriguing in the context of most health research on social capital, which tends to assume that social capital has only health promoting effects. However, previous studies have found that exposure to social situations can provide opportunities for engaging in unhealthy behaviors that tend to occur in social circumstances (i.e., alcohol use) [62]. More frequent interaction with others may also increase the likelihood of experiencing discrimination or other deleterious events that can exacerbate acculturative stress.

Given that neighborhood environments are likely to impact health outcomes through various interrelated mechanisms, the most effective interventions in targeting drinking and driving prevention efforts are likely to be those that can influence behaviors across multiple dimensions. As such, gaining an understating of the neighborhood characteristics that should be targeted as risk and protective mechanisms is essential in the development of effective and culturally-relevant interventions aimed at preventing drinking and driving among Latino immigrants.

## 5. Limitations

Our findings should be interpreted in light of certain limitations. An obvious limitation is our use of respondent-driven sampling (RDS). Although RDS is successful in recruiting hidden populations, such as undocumented immigrants, who make up 22% of the U.S. Latino population [69], it does not ensure a representative sample [48]. Second, although efforts were undertaken to include participants from major Latino subethnicities, some groups (e.g., Mexicans) were not well represented due to their underrepresentation in the Miami-Dade County area in general. Future studies with other Latino subgroups are needed to enhance the generalizability of the results. Third, due to relatively low levels of reported drinking and driving in the sample, various risk behaviors had to be collapsed into one binary variable. Further research is necessary to distinguish the impact of neighborhood level factors in distinct DUI risk behaviors (i.e., riding with an impaired driver versus driving while impaired). Additionally, our study utilized a cross-sectional research design; thus, our findings are correlational and do not establish a causality among the examined variables.

## 6. Conclusions

This study represents an advancement toward understanding the causal mechanisms whereby neighborhood characteristics impact drinking and driving behaviors among Latino immigrants. Our results suggest that future public health interventions should build upon community assets, such as existing levels of social capital, which may improve health in those communities. This knowledge is significant to public health, as it can lead to the creation of impactful and sustainable interventions that can alter the systems that cultivate the disproportionate impact of alcohol-related crashes affecting U.S. Latino communities.

## Figures and Tables

**Table 1:** Sample demographics (*n* = 467).

Variable		*n* = 467	%	
Gender	Female	212	45.4	
	Male	255	54.6	
Documentation Status	Documented	394	84.4	
	Undocumented	73	15.6	
Education	Less than high school	104	22.3	
	High school diploma	234	50.1	
	Some training/college	114	24.4	
	Bachelor’s (4–5 years college)	12	2.6	
	Post graduate/professional	3	0.6	
Region of Origin	Cuba	199	42.6	
	South America	130	27.8	
	Central America	133	28.5	
	Other Caribbean	5	1.1	
DUI Risk Behavior	Yes	61	13.3	
	No	398	86.7	
		Mean (SD)	Skewness	Kurtosis
Age		31.84 (4.97)	−0.24	−1.14
Annual Income		$19,962.52 ($16,524.38)	9.36	137.15
Neighborhood	Informal Social Control	18.18 (4.63)	−0.86	0.97
	Social capital	2.91 (2.70)	2.50	7.28
Social Support		3.73 (1.04)	−0.41	−0.61
Acculturative Stress		3.18 (3.77)	2.44	6.10

DUI: Driving Under the Influence; SD: standard deviation.

**Table 2:** Bivariate correlations of key observed variables.

Variable		1	2	3	4	5	6	7	8
1.	DUI Risk Behavior	1.00							
2.	Informal Social Control	−0.08	1.00						
3.	Social Capital	0.06	0.14 **	1.00					
4.	Social Support	−0.17 **	0.27 **	0.11 *	1.00				
5.	Acculturative Stress	0.08	0.02	0.22 **	−0.08	1.00			
6.	Gender	0.04	−0.10 *	−0.06	−0.19 **	−0.05	1.00		
7.	Documentation Status	−0.00	0.20 **	0.02	0.23 **	−0.04	−0.14 **	1.00	
8.	Alcohol Use	0.22 **	−0.20 **	0.06	−0.23 **	0.11 *	0.25 **	−0.13 **	1.00

Note: * = *p* < 0.05; ** = *p* < 0.01.
